# Neurophysiological Parameters Influencing Sleep–Wake Discrepancy in Insomnia Disorder: A Preliminary Analysis on Alpha Rhythm during Sleep Onset

**DOI:** 10.3390/brainsci14010097

**Published:** 2024-01-19

**Authors:** Francesca Berra, Elisabetta Fasiello, Marco Zucconi, Francesca Casoni, Luigi De Gennaro, Luigi Ferini-Strambi, Andrea Galbiati

**Affiliations:** 1 Department of Psychology, “Vita-Salute” San Raffaele University, 20132 Milan, Italy; francescaberra1997@gmail.com (F.B.); elisabettafasiello91@gmail.com (E.F.); ferinistrambi.luigi@hsr.it (L.F.-S.); 2 IRCCS San Raffaele Scientific Institute, Department of Clinical Neurosciences, Neurology–Sleep Disorders Center, 20132 Milan, Italy; zucconi.marco@hsr.it (M.Z.); casoni.francesca@hsr.it (F.C.); 3 Department of Psychology, Sapienza University of Rome, 00185 Rome, Italy; luigi.degennaro@uniroma1.it; 4 Body and Action Lab, IRCCS Fondazione Santa Lucia, 00179 Rome, Italy

**Keywords:** insomnia disorder, sleep disorder, sleep state misperception, sleep discrepancy, polysomnography, hyperarousal theory, alpha rhythm

## Abstract

Sleep state misperception (SSM) is a common issue in insomnia disorder (ID), causing a discrepancy between objective and subjective sleep/wake time estimation and increased daytime impairments. In this context, the hyperarousal theory assumes that sustained central nervous system activation contributes to the SSM. This study investigates factors influencing SSM during sleep latency (SL) and total sleep time (TST). Objective polysomnographic sleep variables (the alpha density index, latency-to-sleep stages and the first K-complex, and Rapid Eye Movement (REM) arousal density) and subjective sleep indices, taken from sleep diaries, were analyzed in 16 ID patients. Correlation analyses revealed a positive association between the degree of SL misperception (SLm) and the percentage of epochs that contained a visually scored stereotyped alpha rhythm during objective SL. A regression analysis showed that the REM arousal density and alpha density index significantly predicted TST misperception (TSTm). Furthermore, the degree of SLm was associated with an increased probability of transitioning from stage 1 of non-REM sleep to wakefulness during subjective SL. These findings support the role of hyperarousal in SSM and highlight the importance of alpha activity in unravelling the heterogeneous underpinnings of SSM.

## 1. Introduction

Insomnia disorder (ID) is characterized by difficulties initiating or maintaining sleep, accompanied by non-restorative sleep quality and daytime impairments such as fatigue, cognitive impairment, and mood disturbances [1]. The prevalence of ID corresponds to 6% of the population when based on the definition provided by the Diagnostic and Statistical Manual of Mental Disorders, Fifth Edition, Text Revision (DSM-5-TR), which includes a time criterion (i.e., symptoms must be present at least three times a week for at least three months) as a key aspect of an ID diagnosis [2,3,4,5].

One of the most widely accepted models within the scientific community to explain ID pathophysiology is Spielman’s 3P model. This behavioral framework elucidates the progression of ID from acute occurrences to chronic and self-perpetuating states. The model is founded on the interplay between three key components: predisposing, precipitating, and perpetuating factors. The first two types of factors delineate a stress–diathesis conceptualization, explaining how ID emerges initially, where predisposing elements encompass genetic and early life stress factors, contributing to an individual’s vulnerability to ID. Precipitating factors, instead, involve significant events that act as triggers for acute episodes of insomnia. The third type of factor defines how cognitive and behavioral aspects influence and perpetuate the disorder and includes hyperarousal, maladaptive behaviors, and cognitive elements, assuming a pivotal role in sustaining and intensifying ID over time [6,7].

A core feature of individuals affected by ID concerns the estimation of sleep–wake times. Indeed, ID patients tend to overestimate the sleep onset and underestimate the total sleep time (TST) [8]. In some cases, this deficit is so pervasive that it leads to a specific condition previously categorized as ‘Paradoxical Insomnia’, characterized by a phenomenon known as sleep state misperception (SSM). Despite objective sleep recordings being in normal ranges, these individuals report sleeping little or not at all, highlighting a significant discrepancy between subjective and objective estimations [9,10]. The level of distress and the daytime consequences experienced by patients regardless of their objective sleep duration underline the considerable role of SSM in the maintenance of ID symptoms [9]. Therefore, gaining a better understanding of the mechanisms behind this phenomenon would contribute to a deeper understanding of ID itself and facilitate the development of more targeted therapeutic approaches.

The hyperarousal model is the most accredited hypothesis proposed to explain the SSM phenomenon in ID, as it stems from the sustained activation of the central nervous system that leads ID patients to perceive themselves as awake instead of asleep. 

Hyperarousal is defined as a condition characterized by a relatively heightened 24 h (both waketime and nighttime) cognitive, emotional, physiological, and cortical arousal. These factors contribute to expressing symptoms at both subjective and objective levels [11]. Hyperarousal can manifest in separate but interconnected domains, such as increased brain activity, elevated heart rate, and heightened cognitive alertness during bedtime, making it challenging to transition into a state of sleep [11,12]. According to this hypothesis, ID patients demonstrate an elevated frequency of arousals during Non-Rapid Eye Movement (NREM) sleep compared to healthy controls [13,14]. Additionally, during sleep onset and early sleep stages, they show increased electroencephalogram (EEG) fast frequencies (i.e., beta and gamma) usually associated with the waking state [15]. Coherently, a moderate beta activity increase during NREM sleep has been related to the underestimation of TST [16]. However, SSM is also a common phenomenon in the general population, and it is associated with peculiar EEG features. Indeed, also in good sleepers, TST underestimation is associated with higher cortical activation (a reduced delta/beta power ratio) during NREM and REM sleep, and, on the other hand, TST overestimation is associated with the increased EEG activation of slow frequencies during REM sleep [17]. Moreover, in an fMRI study based on ID patients, altered functional connectivity was observed during NREM sleep, specifically in the period immediately following the sleep onset, between cortical and subcortical regions implicated in higher cognitive processes. Specifically, ID patients during NREM sleep showed increased functional connectivity related to brain areas responsible for inwardly directed attention and conscious awareness. The authors posit that such connectivity patterns may be intricately linked to the primary complaints of patients regarding sleep and their corresponding perceptions [14]. 

Even considering sleep as a local phenomenon, recent evidence suggests that individuals with ID exhibit an altered physiological state of arousal. Recent research on local sleep, a term used to indicate the presence of sleep or wakefulness confined to specific brain regions, has reported insightful results [18,19]. A high-density EEG study by Riedner and colleagues revealed that the subjects with ID generally exhibited a greater amount of high frequencies (>16 Hz) and elevated alpha activity in the sensory and sensorimotor areas compared to the healthy controls, even when the brain globally appeared to be in deeper NREM sleep phases [20]. Additionally, the evidence showed that ID patients present increased EEG activation during NREM sleep localized in the mid-posterior brain areas, which correlated with SSM, suggesting that these subjects might be more sensitive to correctly perceiving shifts towards wakefulness-like brain activity rather than misperceiving their sleep [17]. Moreover, the alpha band is also involved in the imbalance of information flow between the anterior and posterior cortex, which significantly decreases during transitions between a state of wakefulness (W) and NREM sleep stage 1 (N1) [21]. 

Moreover, it is known that individuals with ID experience significant sleep fragmentation during the process of falling asleep [22], which is directly associated with the extent of their SSM [23]. This phenomenon could be attributed to insomniacs’ difficulty transitioning from being awake to sleeping. In this context, the alpha rhythm is a brainwave activity particularly relevant during sleep onset, manifesting itself when an individual is awake but with eyes closed, attempting to relax [24]. Remarkably, alpha rhythm alterations have been found to be a characteristic trait in ID patients [25,26]. However, up to now, no study has investigated the relationship between alpha waves during the wake–sleep transition and their impact on SSM both during sleep onset and the entire night. Moreover, the same sleep fragmentation in ID conditions was observed during the whole night. Wei and colleagues, through a sleep stages transition probabilities analysis, revealed a distinctive vulnerability of NREM stage 2 (N2) sleep during the night in insomnia patients. Indeed, the stable N2 sleep in ID patients was more likely to shift to lighter sleep or wakefulness than in the HCs [27].

Another relevant putative neurophysiological mechanism underlying SSM concerns the instability of the REM phase. In fact, it has been observed that ID patients exhibit increased REM instability (i.e., increased REM arousal) [28], which is associated with hyperactivity of the locus coeruleus, a structure responsible for promoting wakefulness [29]. In light of this, due to the similarities in EEG activation between REM sleep and wakefulness, as well as the presence of vivid mental activity often associated with REM sleep, individuals may perceive REM periods with greater fragmentation as wakefulness [30]. Furthermore, individuals with ID identified as higher misperceivers demonstrate more emotional-related symptoms compared to those classified as moderate misperceivers. The elevated levels of daytime consequences in the emotional domain align with the observation of impaired REM sleep in those with SSM, considering the role of REM sleep in emotional regulation [31].

Starting from these premises, this study aimed to determine which polysomnographic (PSG) parameters in ID patients affect the subjective perception of their sleep during the wake-to-sleep transition and throughout the whole night. We aimed to (i) evaluate the impact of the stereotypical alpha rhythm manually scored during the sleep latency (SL) period and the period from latency to the first K-complex (KC) or NREM stage 3 (N3) sleep in accounting for SL misperception (SLm), and (ii) to test the association between TSTm and electrophysiological indices like REM sleep fragmentation. Finally, considering the heightened instability throughout an entire night’s sleep that characterizes individuals with ID [13], we aimed to assess the transition probabilities between sleep and wake stages during subjective SL (SL_subj_) using a Markov chain analysis and to test its impact on SLm.

We hypothesized that (i) an increased amount of alpha rhythm during objective SL (SL_obj_) might be associated with SLm and (ii) increased levels of the REM arousal index might correlate with TSTm. Moreover, we expected an association between sleep instability during SL_subj_ and SLm.

## 2. Materials and Methods

### 2.1. Participants

The study utilized a convenient sample, a pragmatic approach chosen due to the pilot clinical nature of our investigation. Sixteen ID patients (M/F: 12/4; mean age: 51.3 ± 13.34; range: 31–79 years) recruited from the Sleep Disorder Centre of San Raffaele Hospital, Milan (Italy) between November 2019 and November 2021 voluntarily participated in the study. A sleep medicine expert diagnosed ID according to the criteria of the third edition of the International Classification of Sleep Disorders (ICSD-3) [1]. Patients undergoing unstable drug therapy (less than 3 months) with other sleep, neurological, or psychiatric disorders were excluded from the study. 

### 2.2. Polysomnography

The ID patients underwent a nocturnal PSG evaluation in their hospital room. The PSG recording included 6 EEG channels (F3, C3, O1, F4, C4, O2, referred to as the contralateral mastoid) placed according to the international 10–20 system [32]. Moreover, electrooculography (EOG) and electromyography (EMG) of the submentalis and tibialis muscles were carried out, and electrocardiograms (ECGs) and airflow and respiratory effort signals were acquired according to the American Academy of Sleep Medicine (AASM) criteria [20]. The sleep scoring was conducted in 30 s epochs, where each epoch was categorized into different sleep stages, including wake, N1, N2, N3, and REM sleep. Specific criteria, based on a visual inspection of EEG patterns, eye movements, and muscle tone, were used to determine the sleep stage of each epoch. This standardized approach ensures consistency and reliability in sleep stages across different studies [33]. The sleep scoring was performed manually through Polyman Software 1.5 for Windows 10, referring to the standard criteria defined by the AASM Manual for the Scoring of Sleep and Associated Events, Version 2.6 [34].

### 2.3. Sleep Diary

After the PSG recording night, the patients filled in an electronic sleep diary within 15 min after their final awakening. Online delivery allows for an objective assessment of when the compilation process was finished, excluding all patients who submitted their diaries with inadequate timing. The patients were instructed to complete the diary by referring only to their subjective feelings and memories and not to time-tracking devices (i.e., clocks, alarm clocks, electronic devices, etc.) or advice from third parties. The diary included questions about several sleep parameters, such as bedtime (BT), subjective sleep onset time (SO_subj_), SL (in minutes), number of nocturnal awakenings (nAWK), wake after sleep onset (WASO, in minutes), TST (in minutes), final wake-up time, and out-of-bedtime time.

### 2.4. Sleep State Misperception Indices

Two indices were used to evaluate the degree of SSM during specific moments of the night (i.e., sleep onset and the whole night).

**Figure 1:**
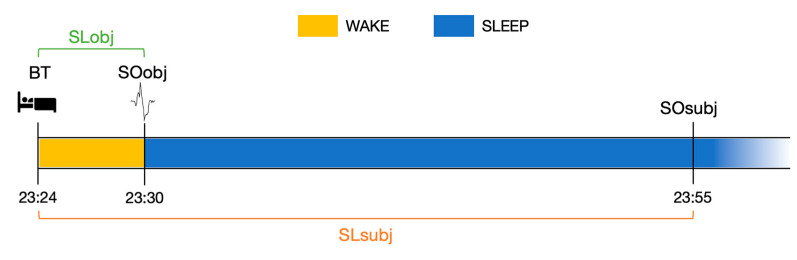
Graphical representation of sleep onset modeling. At the beginning of the graph, the time at which the subject ceased all activities to begin trying to sleep (bedtime, BT) is indicated. Then, the time of objective sleep onset (SO_obj_) and the time of subjective sleep onset (SO_subj_) are indicated. Abbreviations: BT: bedtime, corresponds to the moment when the patient decided to stop any activity to try to fall asleep; SO_obj_: objective sleep onset, corresponds to the appearance of the first K-complex; SO_subj_: subjective sleep onset, indicated by patients in the sleep diary; SL_obj_: objective sleep latency; SL_subj_: subjective sleep latency.

To evaluate SLm, the objective (i.e., extracted by PSG recordings) and subjective (i.e., extracted by sleep diaries) SL indices were compared using the formula (SL_subj_/SL_obj_) × 100, and higher scores (>100%) indicate a greater overestimation of SL. The same calculation was carried out for the entire night, comparing the objective/subjective TST indices: (TST_subj_/TST_obj_) × 100. Lower TSTm scores (<100%) denote higher TST underestimation [17] (Figure 1).

Moreover, we also calculated a raw index of the subjective and objective SL discrepancy (i.e., SL_subj_-SL_obj_), which is useful for splitting the sample into two subgroups (8 participants each) based on the cut-off of 10 min (i.e., <10 min: lower discrepancy; >10 min: higher discrepancy). This threshold was chosen based on evidence by Hermans and co-workers, who determined that a minimum of 10 min of uninterrupted sleep is needed to be recognized as such [23]. Therefore, any discrepancy below 10 min might be attributed to chance.

### 2.5. Electrophysiological Indices

The indices selected from the literature are described in the following paragraphs to determine which EEG parameters can account for the degree of SSM.

#### 2.5.1. Alpha Density

The alpha activity was visually scored on occipital derivations (i.e., O1 and O2) in micro-epochs of 3 s during SL_obj_. To label a micro-epoch as containing stereotyped alpha rhythm (frequency range of 8–13 Hz), this activity must be detected for at least 50% (1.5 s) of each micro-epoch. The alpha density index was computed by dividing the total number of 3 s micro-epochs containing stereotyped alpha rhythm by the total number of micro-epochs during SL_obj_ and expressed as a percentage (%α).

#### 2.5.2. Latency-to-Sleep Stages

The latency-to-sleep stages were calculated by measuring the time elapsed between BT and (i) the occurrence of stage N1, (ii) the first KC, and (iii) the first epoch of N3. The KC was scored according to the definition provided by the AASM, i.e., a waveform characterized by a sharp negative deflection immediately followed by a positive component, with a total duration between 0.5 and 3 s and a peak-to-peak amplitude ≤ 75 µV on frontal derivations (F3 and F4) [34].

#### 2.5.3. REM Arousal Density

The percentage of arousal occurring during REM sleep (the REM arousal density index—A-REMd) was calculated based on the AASM definition. REM sleep arousal was identified when a sudden shift in the EEG frequency to an alpha or theta rhythm, or frequencies exceeding 16 Hz (excluding sleep spindles), occurred. The shift should last for at least 3 s and be accompanied by an increase in submental muscle activity lasting at least 1 s, followed by a period of stable sleep lasting a minimum of 10 s [34].

#### 2.5.4. Sleep Transition Probability

A Markov chain analysis, a statistical method used to model and analyze systems transitioning from one state to another, was performed using the R package Markovchain [35]. This analysis determines the probability of transitioning between different states, and it is independent of how a system arrived at its current state [36]. The Markov chain analysis was carried out to analyze the sleep stage and wake transitions during SL_subj_ and to calculate the transition probabilities between the sleep and wake stages [37]. Markov chains, or Markov processes, are stochastic models describing a sequence of possible events, where the probability of each event depends only on the state attained in the previous event. Markov chains can have a discrete state space or a discrete index set and are widely applied as statistical models for various real-world processes. Our Markov chain analysis was implemented to investigate transitions between different sleep stages and wakefulness during the SL_subj_ period. This analysis allows us to understand how sleep transitions contribute to a subjective perception of the sleep onset process. Markov chains are characterized by a state space, a transition matrix describing the probabilities of specific transitions, and an initial state across the state space. We assumed all possible states and transitions were included in the definition of the process, ensuring there was always a next state and that the process did not terminate. In practical terms, we evaluated the probability of transitioning between sleep states (e.g., wake, N1, N2, N3, REM) during SL_subj_. This methodology may provide new insights into how sleep transitions contribute to subjective perceptions of the falling asleep process. 

### 2.6. Statistical Analysis

The data were analyzed using Statistical Package for Social Sciences (IBM SPSS Statistics for Windows, Version 25.0. Armonk, NY, USA: IBM Corp.), Jeffreys’s Amazing Statistics Program (JASP, version 0.16) [38], and R software version 4.3.2. The descriptive statistics for gender, age, disease duration, drug treatment, PSG, and sleep diary indices were computed. The Shapiro–Wilk normality test was used to assess the sample distribution. Since the data did not meet the normality assumption and given the small sample size, non-parametric tests were performed. 

A stepwise backward multiple linear regression analysis was conducted to assess whether the A-REMd index, the %α index, and the percentage of N3 significantly predicted the TSTm. 

Regarding SLm, since the basic assumptions for multiple linear regression were not met, Spearman’s rho test was performed to evaluate (i) the correlation between SLm, the %α index, and the SL indices (i.e., to N1, to the first KC, and to N3), and (ii) the correlation between the TSTm, A-REMd, %α, and the sleep stages percentages. 

Furthermore, the two subgroups, split based on their degree of SL discrepancy (< or >10 min), were compared for the %α index and the Markov transitioning probability using the Mann–Whitney U test.

Notably, we conducted analyses to assess the potential impact of the presence of stable benzodiazepine therapy on our results. The dichotomy variable ‘presence of therapy’ was included as a covariate in analyses examining between-group differences. Furthermore, we performed stratified correlation analyses in the two subgroups to identify the EFFECT OF the presence of drug therapy.

## 3. Results

### 3.1. Demographical and Clinical Features

Table 1 presents the sample’s (n = 16) demographic and clinical features. The mean ID duration was 13.14 ± 6.62 years (range: 2–22 years), with a male prevalence (n = 12; 75%).

The subjective sleep parameters differed from those recorded by the PSG examination. As expected, the average SL_subj_ period was longer than the SL_obj_ period (U = 186.5; *p*-value = 0.028), and the TST_subj_ was lower than the TST_obj_ (U = 2.1; *p*-value = 0.044). Surprisingly, the subjective WASO was lower than the objective WASO (U = 81; *p*-value = 0.049). 

Ten of the sixteen patients in the sample had been on stable benzodiazepines (i.e., Clonazepam, Delorazepam, or Lormetazepam) therapy for at least 3 months. However, the presence of benzodiazepine intake did not significantly alter the observed outcomes, ensuring that our reported results remain robust and unaffected by the presence of drug therapy.

### 3.2. Linear Regression Analysis

In the stepwise backward multiple linear regression analysis predicting the TSTm, a model initially including A-REMd, %α, and N3 latency was assessed. The final model, however, identified that the two-factor model comprising A-REMd and %α (F = 9.6, *p*-value = 0.003) provided a significantly better fit. This model accounted for a substantial portion of the variance, explaining 59% of the variability in the TSTm. The adjusted R-squared value indicated that the model with A-REMd and %α as predictors was a better fit compared to the three-factor model (adjusted R^2^ = 0.576 vs. 0.539, respectively). The resulting regression line is expressed as follows:

TSTm = 132.67 − 56.04 × (A-REMd) − 178.48 × (%α)

Age did not influence the A-REMd and %α indices.

### 3.3. Relationship between Objective Sleep Indices and the Degree of SSM

**Figure 2:**
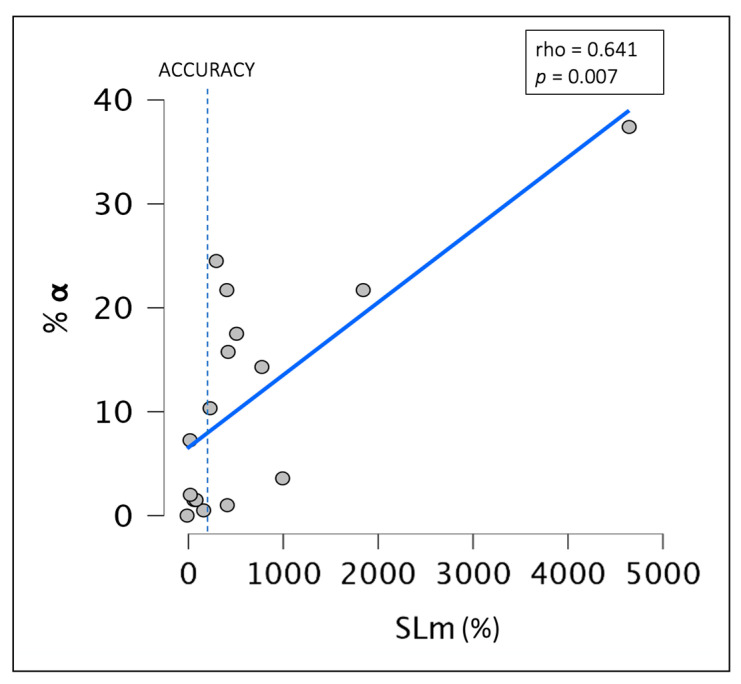
Scatterplot representing the correlation between the %α index during SLobj and SLm. Abbreviations: %α: percentage of alpha rhythm density; SLm: sleep latency misperception.

The correlation analyses showed no significant relationship between N1, N3, the first KC latencies, and SLm. On the other hand, a significant positive correlation was observed between the %α index during the SL_obj_ and the SLm periods (*rho*_Spearman_ = 0.641; *p*-value = 0.007) (Figure 2). 

**Figure 3:**
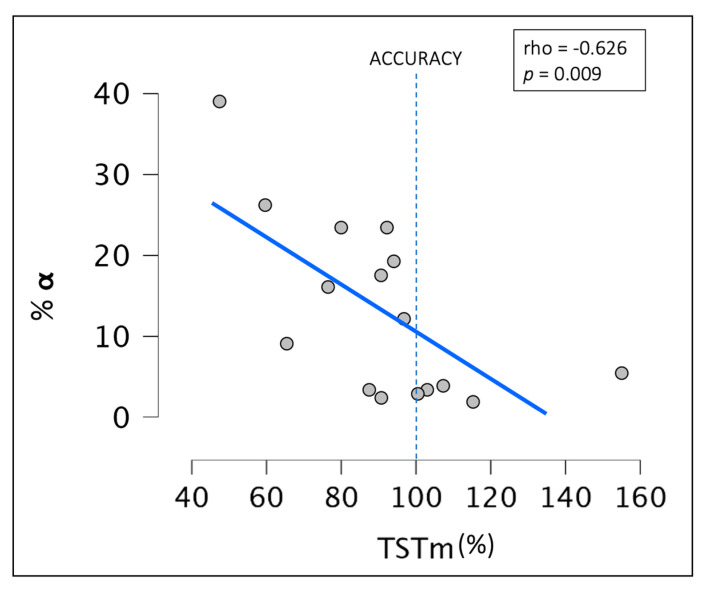
Scatterplot representing the correlation between alpha rhythm density during SL_obj_ and TSTm. Abbreviations: %α: percentage of alpha rhythm density; TSTm: total sleep time misperception.

The correlation analyses showed no significant relationship between the sleep stage percentages, A-REMd, and TSTm. However, a negative correlation was found between the %α index during the SL_obj_ period and the TSTm (rho_Spearman_ = −0.626; *p*-value = 0.009) (Figure 3). It was concluded that age did not affect the results.

### 3.4. Differences between Groups Based on the Degree of Misperception

**Figure 4:**
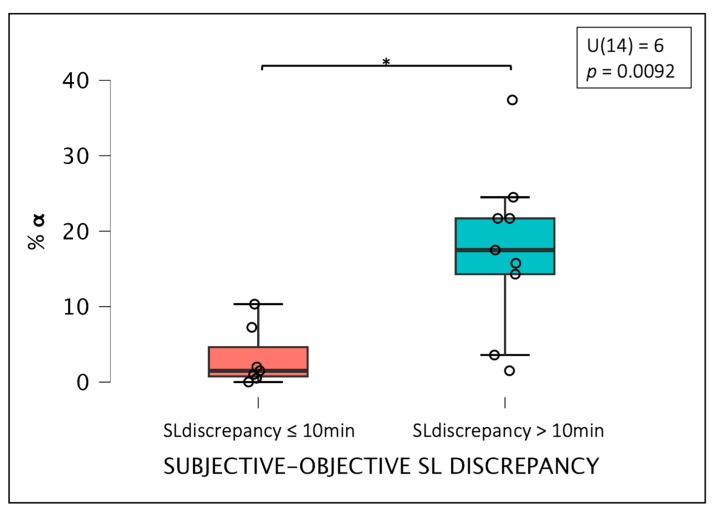
Differences in %α density between the two subgroups categorized based on objective/subjective SL discrepancy, where * indicates the presence of a significant difference between the two groups. Abbreviations: %α: percentage of stereotyped alpha rhythm.

Comparing the groups based on the objective/subjective SL discrepancy (above or below 10 min), a significant between-group difference was observed (U = 6; *p*-value = 0.0092). As measured by the rank-biserial correlation, the effect size was −0.825, suggesting a large effect. The group with a higher SL discrepancy (>10 min) exhibited a higher %α (16.5 ± 10.8) compared to the subgroup with an SL discrepancy of less than 10 min (2.6 ± 3.8) (Figure 4).

### 3.5. Markovian Matrices

**Figure 5:**
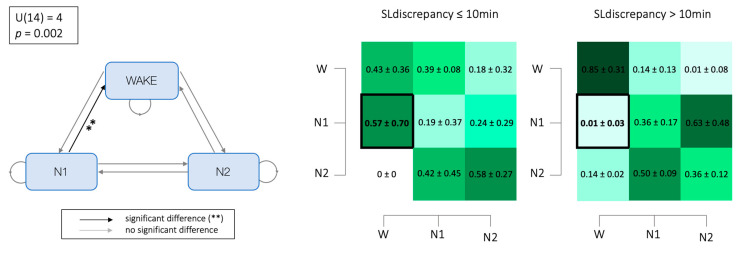
Differences in the transition probability: the left side of the figure illustrates a graphical representation of the mean Markovian matrix, where grey lines indicate non-significant differences in the transition probabilities between states, while black lines marked with ** highlight significant variations in the transition likelihoods between the two groups. On the right side, heat maps show the mean ± standard deviation of the probabilities of transitioning from one state to another for both groups. Significant results, denoted in bold, report the distinctions in the transition dynamics between the groups. The color gradient within the heat maps represents the power of the probability, with darker colors indicating higher probabilities. NB: the absence of transition probabilities involving N3 and REM is attributed to a high proportion of subjects who did not attain these sleep stages during subjective sleep latency. Abbreviations: N1: Non-Rapid Eye Movement sleep stage 1; N2: Non-Rapid Eye Movement sleep stage 2; N3: Non-Rapid Eye Movement sleep stage 3; REM: Rapid Eye Movement sleep. ** indicates significant results (*p* < 0.05).

The Markovian transition matrices showed an increased probability of transiting from the N1 state to the W state during SL_subj_, which is higher for the group with an SL discrepancy higher than 10 min (SL discrepancy > 10 = 0.57 ± 0.7 vs. SL discrepancy ≤ 10 = 0.01 ± 0.034; U = 4; *p*-value = 0.002; rank-biserial correlation = −0.750) (Figure 5). 

## 4. Discussion

This study investigated neurophysiological factors influencing the discrepancy between subjectively and objectively measured sleep in ID patients, distinguishing between the sleep onset period and the whole night. As anticipated, the participants in our sample tended to overestimate SL and underestimate the TST (i.e., SL_subj_ was significantly greater than SL_obj_; the TST_subj_ was significantly less than the TST_obj_). Notably, contrary to expectations, the subjects reported a significant underestimation of WASO. This unexpected finding aligns with the existing literature, highlighting the considerable variability in WASO assessments across different measuring instruments, environments, and even between nights [39,40]. This variability underscores the limitations of WASO as a reliable index for measuring and assessing SSM [39].

Regarding sleep onset, our findings indicate a significant positive association between the %α index during SL_obj_ and the extent of the degree of SLm, indicating that a higher SL overestimation is related to a higher density of alpha activity during the sleep onset period. This may appear counter-intuitive, as alpha activity is typically associated with relaxed wakefulness with closed eyes, and it is crucial for transitioning from wakefulness to sleep [24]. On the other hand, it is well known that the alpha rhythm is also characteristic of light and shallow sleep [41,42,43]. Hence, individuals with ID who spend more time in this state might be more likely to misperceive the process of falling asleep. 

This finding is consistent with the evidence in the literature reporting that the NREM sleep of ID patients is characterized by more cortical activation than in healthy controls. Several studies have reported an increase in alpha [13,44,45,46], sigma [13,44,45], beta [13,17,44,45,46], and gamma [17,46,47] frequencies and a decrease in delta frequencies [17,44,45,46] during an entire night’s NREM sleep [48]. It was also found that ID patients exhibited increased beta frequencies even during diurnal sleep, as observed in multiple sleep latency tests (MSLTs), and those with lower sleep efficiency and longer latency to MLST showed higher beta power also during night sleep [49]. Furthermore, some studies have shown a positive correlation between increased EEG frequencies and SSM [20,45]. Moreover, heightened alpha activity while falling asleep has been more commonly observed across subjects with ID than healthy subjects [26]. However, the specific role of these frequency bands concerning the misperception of falling asleep has not yet been thoroughly explored. In this context, our data suggest that a hyperarousal state may influence the perception of sleep onset. This is supported by the evidence that SLm is associated with a lower delta/beta ratio in SL_obj_ [50]. Additionally, in a paradigm of programmed awakenings (from N1 and N2), erroneously perceiving sleep as a wakeful state was associated with lower relative theta power and higher alpha, beta, and gamma power [51]. 

The link between the SSM during sleep onset and an index of sleep microstructure preceding falling asleep is not the first finding of its kind. Maes and colleagues found an association between a lower delta/beta ratio and SLm, suggesting that an acceleration of pre-sleep EEG might affect the sleep–wake discrepancy during this period. Furthermore, the amount of beta EEG activity during SL_obj_ was associated with a higher density of KCs immediately preceding a sleep spindle. This finding has been interpreted as an attempt to cope with the hyperarousal state with an extra effort to protect sleep continuity [51]. In this way, it is plausible to hypothesize that these individuals might find it challenging to disengage from a fast EEG rhythm towards a lower frequency and thus fall asleep, perceiving longer wakefulness due to sleep effort, a construct known to perpetuate ID [52]. Supporting the hypothesis that there are challenges in disengaging from wakefulness to sleep, evidence from event-related potential studies indicates that individuals with psychophysiological ID exhibit difficulties in inhibiting information processing during sleep onset (i.e., increased N100 and N350 amplitudes and a decreased overall amplitude) [53,54]. Consequently, an increase in the alpha rhythm density might be associated with the effort expended by a subject in attempting to fall asleep in a state of cognitive arousal. 

Since the association between sleep effort and SSM has not yet been investigated in any study, future investigations might test whether the alpha activity density correlates with objective and subjective measures of sleep effort. Indeed, evidence shows that higher levels of sleep effort (measured with the Glasgow Sleep Effort Scale [55]) are associated with increased ID severity [51]. These data fit coherently into the context of Espie and collaborators’ theory of ID, known as the psychobiological inhibition model, based on the A-I-E (Attention, Intention, Effort) pathway. This model suggests that anxiety about sleep and its consequences leads individuals to focus on their explicit intention to sleep and make efforts to fall asleep. This mechanism paradoxically results in the opposite of the desired outcome: the need for perceived control over sleep leads to sleep interruption [56,57]. According to Espie’s theory, individuals with ID try to exert explicit and conscious control over sleep instead of relying on the automatic and involuntary mechanisms that promote and regulate sleep in healthy individuals. Therefore, the density of alpha activity may be symptomatic of insomniacs’ unsuccessful attempts to achieve a state of forced relaxation leading up to sleep. Consequently, it may be difficult for subjects to disengage from the awake state due to their efforts to sleep [45]. Indeed, when so much attention is focused on sleep and all its related worries, as well as the effort to relax, the subject reaches a state of increased cognitive and physical activation. 

Finally, the Markovian chain analysis revealed higher states instability associated with increased SLm. Specifically, compared to previous evidence in ID [27], the ID patients in our study demonstrate a higher probability of returning to wakefulness while in the N1 sleep stage. This finding again emphasizes the association between SSM and the instability of the sleep system, a relationship observed in previous studies [23,58]. Furthermore, this evidence seems to suggest that a higher amount of alpha activity, characteristic of the waking state with closed eyes [24], might play a central role in explaining the mechanism underlying SSM in individuals with ID. The increased alpha density index at sleep onset observed in the patients that significantly overestimated their SL_obj_ is consistent with their subjective tendency to be anchored to a wake state in this period rather than maintaining N1 sleep or transitioning into deeper sleep stages. 

The correlation analyses revealed no significant results concerning the SSM during the entire night. However, the multiple regression analysis showed that a two-factor model, including the %α during sleep onset and REM arousal density, significantly explained the largest portion of the variance in the TSTm. We hypothesize that its association with the alpha density could be due to the influence of this rhythm on the perception of sleep onset. However, if the increase in alpha activity detected at sleep onset was consistent throughout the night, the finding could align with studies reporting a positive correlation between the amount of alpha rhythm and the TSTm [44,45]. The association between the underestimation of the TST and the REM arousal density is consistent with previous evidence indicating an increase in REM sleep instability in relation to the extent of SSM [30,59] and its association with lighter REM sleep in individuals with SSM [60]. Accordingly, Feige and colleagues (2008 and 2023) reported that the REM arousal index was significantly higher in the subjects with ID compared to the healthy controls, while the arousal index in N2 did not differ between the two groups. These findings could support the involvement of REM sleep in the hyperarousal state, suggesting a relationship between increased arousal during this phase and the degree of SSM [28,61]. Additionally, this aligns with the results obtained by Castelnovo and colleagues, who, in a study involving ID patients categorized into high misperceptor (HM) and moderate misperceptor (MM) groups, identified that those in the HM group reported a reduced frequency of dream recall than those in the MM group. This was observed despite comparable amounts of REM sleep between the two groups, even though the HM group exhibited a significantly higher number of awakenings per hour of sleep [31]. Although REM sleep can be considered a deep sleep state, it is characterized by patterns of fast frequencies of brain activity reminiscent of waking states [60]. Since REM sleep is the stage during which dreams occur most frequently, according to the hypothesis proposed by Riemann and collaborators, the presence of mental contents similar to wakefulness, combined with fragmented sleep, could make these contents more accessible to memory, leading individuals to perceive a period of REM sleep with fragmented dreams due to continuous micro-awakenings as a single episode of wakefulness [30].

## 5. Conclusions, Limitations, and Future Directions

Our results identified two electrophysiological sleep indices related to the SSM phenomenon: the first concerns the amount of alpha activity observed during SL_obj_, and the other is the REM sleep fragmentation; both are ascribable to a hyperaroused state.

Some limitations should be considered in our study. The small sample size and the lack of a control group limit the generalizability of our findings. Moreover, the presence of pharmacologic therapy in most patients could represent a confounding factor. Nevertheless, our results did not show any specific effects of therapy on SSM. Moreover, a meta-analysis conducted by Zhao and colleagues in 2021 revealed a significant impact of benzodiazepine use exclusively on theta activity during wakefulness [46]. Furthermore, we did not investigate cognitive and physiological levels of pre-sleep arousal and levels of sleep effort that might be useful for a more in-depth explanation of our results. A further criticism that may arise is the use of Markovian matrices applied to the sleep system. Indeed, this analysis is only applicable to systems that possess the Markovian property, defined as a property whereby the states of a system are independent of each other, so the term ‘Markovian’ is often replaced by the term ‘memoryless’. Sleep is not a system with independent states, as the probability of transitioning from one stage to another is influenced both by the previous stage and by the time of the night in which we find ourselves [62]. However, the analysis was only applied to the wake-to-sleep transition, which is characterized by intrinsic instability, with the implicit assumption that, at least in this phase and in a pathologic condition such as ID, sleep behaves as a system with independent states. In addition, it is worth noting that the scoring of the sleep stages and indices was performed manually. While this method is widely accepted in the sleep medicine community, manual scoring introduces potential variability [63]. Despite these limitations, studies have demonstrated good intersubjective reliability rates in manual sleep scoring, especially concerning wakefulness and REM epochs [33]. Nonetheless, using automated scoring systems could further enhance the reliability of sleep scoring, and this limitation should be considered when interpreting our results.

Future studies should replicate the present results using a larger sample size, comprising a healthy control group and patients not undergoing pharmacological therapy. Moreover, high-density EEG would enable the consideration of topographical aspects, such as local sleep. Additionally, including rating scales to assess the presence of perceived arousal would facilitate the assessment of the relationship between other sleep-related variables (such as sleep effort) and the misperception phenomenon.

In summary, these findings suggest that an underlying hyperarousal state may play a central role in shaping the discrepancy between how individuals perceive their sleep and the quantitative measurements of their actual sleep patterns. These insights contribute to our understanding of the multifaceted nature of ID and may inform potential strategies for its management and treatment.

## Figures and Tables

**Table 1:** Patients’ demographic and sleep characteristics.

	**n = 16**	**%**			
Male	12	75%			
On medication	10	62.5%			
	**Mean** (Mean ± SD)				
Age (years)	51.3 ± 13.34				
Disease duration ^a^ (years)	13.14 ± 6.62				
	**Objective parameters** (Mean ± SD)	**Subjective parameters** (Mean ± SD)	**U-test**	***p***	**Effect Size** (rank-biserial correlation)
SL (min)	11.69 ± 10.55	34.12 ± 32.60	186.5	**0.028**	**−0.926**
TST (min)	417.12 ± 65.92	379.37 ± 129.12	2.1	**0.044**	**−0.768**
WASO (min)	58.91 ± 47.63	37.12 ± 46.44	81	**0.049**	**−0.562**
ℷN1 (min)	15.66 ± 14.97	--			
ℷN3 (min)	60.09 ± 54.44	--			
ℷK-Complex (min)	26.73 ± 18.22	--			
Alpha density (%)	11.28 ± 0.11	--			
REM arousal density (%)	0.38 ± 0.19	--			
SLm (%)	753.41 ± 1240.56	--			
TSTm (%)	90.99 ± 26.30	--			

Abbreviations: SL: sleep onset Latency; TST: total sleep time; WASO: wake after sleep onset; ℷN1: latency to stage-one sleep; ℷN3: latency to stage-three sleep; ℷK-Complex: latency to the first K-Complex. ^a^ Only 14 out of 16 had disease duration information. Significant results (*p*-value < 0.05) are in bold.

## Data Availability

The conditions of our ethics approval do not permit public archiving of the anonymized study data. Readers seeking access to the data should contact the corresponding author. Access will be granted to named individuals in accordance with ethical procedures governing the reuse of sensitive data. Specifically, requestors must complete a formal data-sharing agreement.
